# Utilization of Smartphones and Attitude Regarding Their Use at the Workplace by Nurses: A Cross-Sectional Study

**DOI:** 10.7759/cureus.57000

**Published:** 2024-03-26

**Authors:** Rakhi Gaur, Vipin Patidar, Suman Kumar, Suresh K Sharma, Vasantha C Kalyani, Nipin Kalal, Shiv K Mudgal

**Affiliations:** 1 College of Nursing, All India Institute of Medical Sciences, Deoghar, Deoghar, IND; 2 Department of Microbiology, All India Institute of Medical Sciences, Deoghar, Deoghar, IND; 3 College of Nursing, All India Institute of Medical Sciences, Jodhpur, Jodhpur, IND

**Keywords:** workplace, utilization, smartphone, nurses, attitude

## Abstract

Introduction: The use of personal smartphones in healthcare settings is widespread, with nurses often integrating these devices into their profession's practices. This study addresses the utilization of and attitudes toward smartphone use among nurses in the workplace.

Methods: This study uses a descriptive cross-sectional design and includes 258 nurses from various departments in a tertiary care teaching and research hospital in India. Data was collected using Google Forms (Google LLC, Mountain View, California, United States) through a socio-demographic questionnaire and two scales: "Utilization of Smartphones at Workplace" and "Attitude Regarding Smartphone Use at Workplace."

Results: The findings indicate that a significant proportion (64.3%) of participants use their smartphones for less than an hour at the workplace. Notably, a substantial majority (78.7%) agree that employers should implement a policy regarding smartphone use. Additionally, 34.1% use their smartphones for personal reasons for less than an hour, with 24.4% specifically engaging in social media use with a statistically significant difference (p <0.05).

Conclusion: The study finds an enormous gap in nurses' smartphone use, with a greater emphasis on personal and social media activities than professional responsibilities. Establishing a collaborative regulatory framework is essential for aligning smartphone use with patient care obligations and ensuring a balance between possible benefits and risks.

## Introduction

Cell phones have been widely used in personal as well as professional contexts since they were first introduced around 40 years ago and now the internet and mobile technology have advanced significantly over the past 10 years, and this has had an influence on healthcare delivery as well [1]. Among mobile technologies, smartphones' quick development is especially noteworthy. These gadgets are portable computers that enable information access anytime, anyplace, in addition to the features often associated with a mobile phone [2]. Through the creation of health applications that cover a wide variety of issues in the health sciences, they have evolved into tools for social network interaction and participation as well as instruments to assist point-of-care decision-making [3].

The usage of smartphones in the healthcare industry is growing, and healthcare professionals (HCPs) are using them more often [4]. Over time, cell phones have grown ubiquitous in the medical field. Approximately 87% of HCPs use a smartphone or tablet at work [5]. Similar to the general public, smartphones are becoming indispensable in the healthcare sector for a variety of reasons, including easier access to online medical information, interaction with colleagues, and patient monitoring. In particular, the smartphone has transformed clinical practice, as seen by improvements in hospital workflow and communication [6].

Health organizations and nursing professionals throughout the globe are looking for ways to use mobile technology to enhance care. The use of smartphones by nurses in the workplace provides benefits such as enhanced communication and access to information, which improve overall patient care. However, the extent and manner in which mobile devices are utilized remain mostly undocumented. In addition, a number of studies have brought up some drawbacks to the usage of smartphones, including distraction and preventing infections [7-10]. Furthermore, there are several issues about patients' privacy being compromised and unethical behavior while using smartphones to discuss medical information [11]. Also, handling possibly contaminated smartphones after hand washing may raise the risk of nosocomial infection through cross-contamination [12].

Despite their widespread use, there is a noticeable paucity of research on HCPs' utilization of smartphones and their attitudes toward smartphone use in the clinical context, particularly among nursing professionals. In order to better understand this, the present study assesses (a) the utilization of smartphones in the workplace and attitude of smartphone users, and (b) the association between utilization of and attitude regarding smartphone use with socio-demographic data. In this context, "smartphones" refers to any of the several kinds of mobile phones that research participants used, all of which had text messaging, voice communication, internet access, and data storage features.

## Materials and methods

Study design and settings

This was a cross-sectional descriptive-analytical study conducted at All India Institute of Medical Sciences, Deoghar, India, from January to November 2023. The study included and focused on nurses working in all departments of a tertiary care teaching and research hospital in India. The study received ethical approval from the Institutional Ethics Committee, AIIMS, Deoghar, India (approval number: AIIMS-DEO/RC-IEC-Fullcommittee/2023-Jan/28). The purpose and protocol of the study were presented to the participants. Consent ensured voluntary involvement, anonymity, and data confidentiality.

Participants

A simple random sampling strategy was used to include male and female nurses who were working at a tertiary care hospital in India. The research encompassed nursing professionals across morning, evening, and night shifts, able to converse in English, demonstrating a voluntary willingness to engage in the study, and possessing a requisite service experience of at least three months. Exclusions from the study were applied to nurses unavailable for data collection during the specified period for any given reason.

Sample size

In order to estimate the number of participants required, the sample size was calculated using the formula (N=n/1+ne2), and 230 was found at the 5% level of significance. A 20% dropout rate was taken into account [13] due to incomplete surveys and withdrawals and to compensate for this, 276 surveys were distributed. Of these, 18 nurses declined to participate in the research, so they were left out and we received 258 responses, with a response rate of approximately 94%.

Data collection procedures and tool

The data were gathered between May 16 and July 20, 2023, through Google Forms (Google LLC, Mountain View, California, United States), which included a socio-demographic questionnaire and two scales on “Utilization of smartphones at the workplace” and “Attitude regarding smartphone uses at the workplace”. The questions on the utilization of smartphones in the workplace in a clinical setting consisted of 10 items on a five-point rating scale where never =1 and always=5. The scale was used to determine how common and often nurses used smartphones while at work. The questions on the attitude regarding smartphone usage at the workplace in a clinical setting consisted of 15 items further classified into two categories: agree and disagree. The researchers developed these scales and experts in the field of nursing validated both the research tools. Cronbach's alpha determined the research tool's reliability to be 0.83 and 0.87, respectively. The investigators created the questionnaire and sent it via WhatsApp (Meta Platforms, Inc., Menlo Park, California, United States) and email to randomly selected participants. Before collecting data, participants were asked to complete a consent form using Google Forms.

Statistical analysis

The data was analyzed using IBM SPSS Statistics for Windows, Version 23.0 (Released 2015; IBM Corp., Armonk, New York, United States). The researcher developed a master data sheet to compute the data and used both descriptive and inferential statistics to calculate the results based on the objectives and hypothesis of the study. The demographic baseline data, which included sample characteristics, was evaluated with frequency and percentage. The chi-square test with a significance threshold of 0.05 was employed to examine the association for categorical variables.

## Results

Socio-demographic characteristics and utilization of smartphone

The sociodemographic outcomes (Table 1) of this study among 258 nurses revealed that 59.3% of participants were aged less than 30 years, 56.6% were male, and 49.2% of nurses were residents of an urban area. The majority of the nurses were assigned to general wards (31.8%), and had one to three years of clinical experience (34.1%). A majority of respondents (65.9%) reported having medical applications on their smartphone, and 62.8% claimed they utilized their phone while working in a clinical environment for personal activities such as social media, internet browsing, gaming, watching movies, listening to music, and making phone calls.

**Table 1:** Frequency distribution of participants in terms of their personal characteristics (N=258). GNM: general nursing and midwifery; HDU: high dependency unit; OT: operation theatre

Socio-demographic categories		Number (percentage)
Age (mean±SD: 30.97±6.627)	Less than 30 years	153 (59.3%)
	31-40 years	77 (29.8%)
	More than 40 years	28 (10.9%)
Gender	Male	146 (56.6%)
	Female	112 (43.4%)
Residence	Urban	127 (49.2%)
	Semiurban	69 (26.7%)
	Rural	62 (24%)
Professional Qualification	GNM	48 (18.6%)
	B.Sc. Nursing	165 (64%)
	M.Sc. Nursing and above	45 (17.4%)
Working Experience	Less than 1 year	51 (19.8%)
	1-3 years	88 (34.1%)
	3-6 years	61 (23.6%)
	More than 6 years	58 (22.5%)
Area of working	General wards	82 (31.8%)
	HDUs/ICUs	48 (18.6%)
	Trauma/emergencies	33 (12.8%)
	Specialized units	47 (18.2%)
	OT	23 (8.9%)
	Any other area	25 (9.7%)
Medical applications in smartphone	Yes	170 (65.9%)
	No	88 (34.1%)
Reason for using smartphone during clinical practice	Personal uses (social sites, surfing the internet, gaming, movie, music, and voice calls)	162 (62.8%)
	Professional use (Educational reading, procedure, drug, etc.)	96 (37.2%)

**Figure 1:**
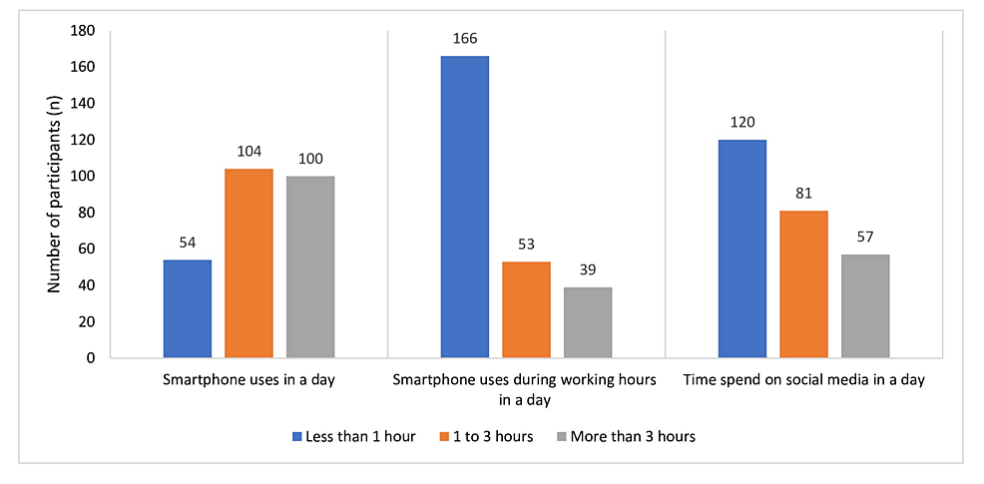
Utilization of smartphone uses in terms of frequency distribution (N=258).

The majority of study participants (n=104; 40.31%) used smartphones for one to three hours a day, and 46.5% (n=120) spent less than an hour on social media (Figure 1). Of the total participants, 64.3% (n=166) used their smartphones for less than an hour at the workplace.

The utilization of smartphones in the workplace in clinical settings among nurses was analyzed and the statement-wise frequency distribution is presented in Table 2. The result revealed that nearly two-thirds of participants (n=159, 61.7%) sometime-to-always used their smartphone as a tool for communicating and coordinating among the medical providers across the hospital, around two-thirds of participants (n=168, 65.1%) sometime-to-always used their smartphone to send messages/emails to colleagues containing patient data, more than half of the participant (n=133, 51.6%) sometime-to-always used their smartphone for personal matters during the clinical hours, approximately half of the participants (n= 121, 46.9%) were sometime-to-always distracted by other colleague’s use of their smartphone during patient care or rounds. and more than one-third of participants (n=98, 38%) sometime-to-always missed an important piece of clinical information because of distraction by smartphone use.

**Table 2:** Statement-wise frequency distribution of participants in terms of their utilization of smartphone during working hours (N=258).

Statement	Never	Rarely	Sometime	Often	Always
	n (%)	n (%)	n (%)	n (%)	n (%)
Used smartphones as a tool for communicating and coordinating among medical providers across the hospital	33 (12.8)	66 (25.6)	83 (32.2)	28 (10.9)	48 (18.6)
Accessed the Internet to find answers to queries about drugs	50 (19.4)	49 (19.0)	80 (31.0)	30 (11.6)	49 (19)
Smartphone use improves patient care and safety	49 (19.0)	41 (15.9)	92 (35.7)	37 (14.3)	39 (15.1)
Used smartphones regularly as a calculator for medication doses	42 (16.3)	53 (20.5)	83 (32.2)	48 (18.6)	32 (12.4)
Used smartphones to send messages/emails to colleagues containing patient data (e.g., their reference number, bed number, medical record number, etc.)	48 (18.6)	42 (16.3)	83 (32.2)	47 (18.2)	38 (14.7)
Surfed the Internet to search for information about clinical references.	30 (11.6)	51 (19.8)	83 (32.2)	55 (21.3)	39 (15.1)
Used smartphones for personal matters (calling, sending messages, assessing social networks, etc.) during clinical hours	51 (19.8)	74 (28.7)	74 (28.7)	33 (12.8)	26 (10.1)
Answered/made phone calls and/or sent messages or emails from mobile during patient rounds	103(39.9)	49 (19.0)	62 (24.0)	23 (8.9)	21 (8.1)
Distracted by other colleagues' use of their smartphone during patient care or rounds	76 (29.5)	61 (23.6)	71 (27.5)	26 (10.1)	24 (9.3)
Missed an important piece of clinical information because I was distracted by my smartphone.	96 (37.2)	64 (24.8)	48 (18.6)	32 (12.4)	18 (7.0)

Attitude regarding smartphone use during working hours

Nurses' attitudes toward smartphone usage at the workplace in a clinical setting have been studied, and the frequency distribution is shown in Table 3. The result indicates that the majority of participants (81.4%) agreed that taking advantage of the opportunities provided by smartphones significantly contributes to more efficient work, three-fourths of participants (75.2%) agreed that forgetting their smartphone makes them feel like they are missing something, around two-third participants (65.1%) agreed that work shifts are really long and sometimes it’s necessary to use a smartphone for things other than work. Similarly, 63.2% of participants agreed that always touching their mobile is a source of infection and the majority of participants (78.7%) agreed that employers should implement a policy on the use of smartphones in the workplace.

**Table 3:** Statement-wise frequency distribution of participants in terms of attitude regarding smartphone uses during working hours (N=258).

Statement	Agree	Disagree
	n (%)	n (%)
Taking advantage of the opportunities provided by smartphones (chat, e-mail…) significantly contributes to more efficient work.	210 (81.4)	48 (18.6)
There is a need for policies regarding the restricted use of smartphones during patient care or rounds.	218 (84.5)	40 (15.5)
Forgetting my smartphone makes me feel like I’m missing something.	194 (75.2)	64 (24.8)
I’m always looking at my smartphone which disturbs me during patient care.	150 (58.1)	108 (41.9)
Work shifts are really long and sometimes it is necessary to use smartphones for other things than work.	168 (65.1)	90 (34.9)
I’ve used it to look up medications that I wasn’t exactly familiar with or didn’t know how to administer them, to calculate doses.	194 (75.2)	64 (24.8)
Simply having my smartphone with me makes me feel safer and more relaxed.	199 (77.1)	59 (22.9)
Always touching my mobile, after which I touch my face or touch the patient, is a source of infection.	163 (63.2)	95 (36.8)
Measures should be taken to regulate the use of smartphones.	189 (73.3)	69 (26.7)
I feel I should switch off my smartphone during working hours.	164 (63.6)	94 (36.4)
If the employer strictly bans the use of smartphones, it will have a negative impact on my morale.	189 (73.3)	69 (26.7)
Smartphone is the best option for keeping in touch with patients, colleagues, and supervisors.	199 (77.1)	59 (22.9)
Unproductive use of smartphones like calls to friends and family during working hours will be harmful to organizational performance.	193 (74.8)	65 (25.2)
Personal phone calls should be during breaks and lunchtime only.	203 (78.7)	55 (21.3)
Employers should implement a policy on the use of smartphones during working hours.	203 (78.7)	55 (21.3)

Categorical association with demographic variables

The data related to the association between the reason for smartphone use during clinical hours and other demographic factors among nurses is shown in Table 4. A total of 81 participants (31.4%) under the age of 30 years reported using their smartphone for personal reasons while at work. It is noteworthy that this number declines with age, with a statistically significant difference (p = 0.000). Further, a statistically significant difference (p = 0.000) was seen as 36% of nurses admitted to using their smartphone for personal reasons while at work, despite having medical applications in the smartphone. In fact, the reason why nurses use their smartphones during work hours is associated with how frequently they use them, as 34.1% of participants used their smartphones for personal reasons for less than an hour. In particular, 24.4% of participants revealed that they used their smartphone for personal reasons, specifically social media, for less than an hour while at work. It is noteworthy that these numbers decrease with time spent in patient care, with a significant difference (p = 0.000 and 0.002, respectively).

**Table 4:** Categorical association (chi-square) of participants in terms of reason for using smartphone during clinical hours with their demographic data (N=258). GNM: general nursing and midwifery; HDU: high dependency unit; OT: operation theatre

Categories		Reason for using smartphone during clinical practice			
		Personal Use	Professional Use	Chi-square value	p-value
		n (%)	n (%)		
Age	<30 years	81 (31.4)	72 (27.9)	16.811	0.000
	31-40 years	57 (22.1)	20 (7.8)		
	>40 years	24 (9.3)	4 (1.6)		
Gender	Male	91 (35.3)	55 (21.3)	0.31^a^	0.861
	Female	71 (27.5)	41 (15.9)		
Residence	Urban	73 (28.3)	54 (20.9)	3.021^a^	0.221
	Semi-urban	47 (18.2)	22 (8.5)		
	Rural	42 (16.3)	20 (7.8)		
Professional Qualification	GNM	35 (13.6)	13 (5)	2.663^a^	0.264
	B.Sc. Nursing	99 (38.4)	66 (25.6)		
	M.Sc. Nursing and above	28 (10.9)	17 (6.6)		
Working Experience	<1 year	30 (11.6)	21 (8.1)	2.008^a^	0.571
	1-3 years	52 (20.2)	36 (14)		
	3-6 years	40 (15.5)	21 (8.1)		
	More than 6 years	40 (15.5)	18 (7)		
Area of working	General wards	49 (19)	33 (12.8)	2.580^a^	0.764
	HDUs/ICUs	32 (12.4)	16 (6.2)		
	Trauma/emergencies	24 (9.3)	9 (3.5)		
	Specialized Units	29 (11.2)	18 (7)		
	OT	14 (5.4)	9 (3.5)		
	Any other area	14 (5.4)	11 (4.3)		
Presence of medical applications in the smartphone	Yes	93 (36)	77 (29.8)	13.944	0.000
	No	69 (26.7)	19 (7.4)		
Smartphone use in a day	<1 hour	35 (13.6)	19 (7.4)	1.297^a^	0.523
	1-3 hours	61 (23.6)	43 (16.7)		
	>3 hours	66 (25.6)	34 (13.2)		
Smartphone use at workplace	<1 hour	88 (34.1)	78 (30.2)	19.128	0.000
	1-3 hours	42 (16.3)	11 (4.3)		
	>3 hours	32 (12.4)	7 (2.7)		
Time spent on social media	<1 hour	63 (24.4)	57 (22.1)	12.328	0.002
	1-3 hours	54 (20.9)	27 (10.5)		
	>3 hours	45 (17.4)	12 (4.7)		

## Discussion

Over the past 10 years, smartphone technology has grown significantly in favor of personal as well as professional reasons [9], to the point where it is now seen as an indispensable tool in the delivery of healthcare [2,7]. Previous studies on healthcare workers, such as nurses and students, conducted since 2015 [3,7] show that most participants use smartphones in clinical settings for both personal and professional purposes. This study, which aimed to determine the utilization of smartphones and the prevalence of and attitudes regarding smartphone use during working hours among nurses, shows that more than half of the participants were male and under 30 years of age, which is consistent with studies by Hitti et al. [14] and Zarandona et al. [15].

Notably, the uniformity of these findings across several studies may indicate a trend or pattern in the demographic traits of study participants. Two-thirds of the respondents stated their smartphone featured medical applications, indicating a significant utilization of medical applications which was consistent with previous studies [14,16]. This is probably due to the complexity of modern healthcare and the growing need for tools by nursing professionals to help them integrate the rapidly evolving literature on contemporary clinical management. As per findings, nurses used their smartphones extensively for personal purposes during working hours for social media, gaming, internet browsing, movie watching, music listening, and phone calls. To be more precise, our findings indicate lower levels of personal usage in clinical settings as compared to other studies [14,15,17]. However, regardless of the reported way of use, the personal uses of smartphones are alarming, especially in the healthcare context where a growing corpus of research highlights the negative effects of disruptions and distractions on patient care. In our study, more than one-third of participants reported using their smartphones for one to three hours per day with the majority only spending an hour or less on social media, and around two-thirds using their smartphones for less than an hour during working hours. This figure is congruent with studies [18-20] that demonstrated the time spent on smartphones was more with accessing social networks was the main use of smartphones. This could suggest that nurses are mindful of how they use their smartphones while at work and may put patient care and professional obligations ahead of prolonged personal smartphone use.

In reference to the utilization of smartphones while at work, the study findings revealed that the majority of participants sometimes utilized their smartphones for a variety of healthcare-related tasks, including communicating with other medical professionals, using a medication calculator, and obtaining clinical references. These findings are consistent with earlier studies [21-23]. The uniformity of the outcomes highlights how smartphones might facilitate better coordination, communication, and access to clinical information in the realm of medicine. It suggests that smartphones are widely recognized as helpful tools in healthcare, enhancing the efficiency and connectivity of healthcare providers for tasks ranging from messaging to acquiring critical medical information.

The widespread effects of smartphone use on social interactions and daily routines are becoming more widely acknowledged, as are the drawbacks, most notably distractions. Unexpectedly, the study's findings provide a rather optimistic picture in which a significant number of participants never replied to emails or messages while on patient rounds, never overlooked important clinical details because of smartphone distraction, and never encountered being distracted by a colleague's smartphone use, while a small percentage of results indicate distraction. These findings imply that healthcare providers may be skillfully controlling their smartphone use in this particular setting to minimize distractions and keep their attention on vital clinical duties while providing patient care. However, this is in contrast to previous studies [15,19,24], which have shown that nurses' and students' frequent smartphone usage during clinical practice might lead to distractions. Thus, it is possible to conclude that, although smartphone distraction is acknowledged as a problem in healthcare, there are situations in which nurses utilize smartphones in a way that is disciplined in order to guarantee that patient care always comes first.

Regarding the attitudes of nurses on the use of smartphones while at work, the findings of the current study provide a nuanced viewpoint on using smartphones at work, especially in healthcare settings. Although two-thirds of participants acknowledged that they effectively use smartphones to improve work efficiency, a significant proportion (two-thirds) of participants agreed that regulations prohibiting the use of smartphones during rounds or patient care are necessary, and measures to regulate smartphone use during working hours are important. This implies that participants are aware of the possible advantages of using smartphones for work-related tasks, but they are also aware that regulatory measures are necessary to keep the setting focused and distraction-free during important healthcare procedures. The use of smartphones in healthcare settings has to be regulated, despite the fact that they provide many potential benefits for the healthcare sector [25]. It is challenging and often controversial to outright forbid smartphone use in healthcare settings. The delicate nature of such regulations is revealed by more than two-thirds of study participants who said they believed that a ban on smartphone use during work hours would negatively affect their morale. However, prior studies indicate that healthcare organizations must develop a plan to enhance knowledge about ethical smartphone usage and encourage its professional application instead of a complete prohibition [26].

The research population appears to have a high emotional attachment to or reliance on smartphones, as seen by the majority of participants reporting that leaving their smartphone makes them feel as though they've missed something. Meanwhile, more than half of respondents claim that long work shifts make it necessary to occasionally use a smartphone for non-work-related activities. Despite the lack of prior research to corroborate or contradict the study's findings, this dual viewpoint highlights the significance of comprehending the practical and emotional aspects of smartphone use in the workplace, pointing to the need for complex policies that strike a balance between productivity and connectivity as well as well-being and focus during working hours.

Another major challenge in a healthcare setting is controlling infections and prevention. The study results indicate that more than half of the participants agree that touching patients or their phones often may increase the risk of illness transmission. This awareness emphasizes the requirement of good hygiene practices, particularly in relation to smartphone use, and is in line with the significance of infection control in hospital settings. Furthermore, the fact that the majority of participants said they believed it was best to switch off their smartphones while at work shows that nurses are open to thinking about ways to reduce the hazards that may arise from using smartphones. This might be an indication of a proactive approach to reducing outside distractions, keeping the patient's needs front and center, and following infection control procedures.

The results of the current study show significant age-related variations in smartphone use in healthcare settings during working hours. Individuals over the age of 40 years were less likely than those under the age of 30 to use their smartphones for personal purposes. The statistically significant difference in a decline in personal smartphone use with age points to a possible generational gap in smartphone usage among nurses. The present findings are consistent with earlier research [21], which also indicates that acknowledging these generational disparities may guide the formulation of customized training initiatives and guidelines aimed at addressing the diverse requirements and customs around smartphone usage in healthcare settings. The study also indicates a relationship between the reasons why nurses use their smartphones during work hours and how frequently they do so. In particular, one-third of participants spent less than an hour using their smartphones for personal purposes while the majority of them admitted to spending less than an hour at work on their smartphones, mostly for social media. Most importantly, these percentages show significant variations. While no prior research has been conducted to support or contradict the study's findings, the results suggest that when their patient care obligations are lighter, nurses could be more prone to use their smartphones for personal purposes. This data can help shape policies intended to control smartphone usage in healthcare facilities, possibly taking workload and patient care responsibilities into account. Maintaining an exact distinction between personal and professional smartphone use among healthcare professionals may be challenging, as evidenced by the statistically significant difference showing one-third of nurses acknowledged using their smartphones for personal reasons while at work, even though their smartphones had medical applications installed. Although there hasn't been any earlier research to support or refute the study's findings, it suggests that having medical applications on a smartphone might not be enough to prevent nurses from using them for personal use while at work. It highlights the significance of all-encompassing approaches to promote appropriate smartphone usage in hospital settings, including explicit guidelines, instruction, and even technology solutions.

Limitations

The findings of our study should be considered in the context of its limitations. The study acknowledges the limitations associated with the cross-sectional design. However, the authors posit that this design was deliberately selected to attain a balance between safeguarding privacy, assuring a high response rate, and upholding the study's validity within its specific context. Another limitation underlines the possible effect of social desirability bias, even with assurances of response confidentiality from the research team. The bias poses a challenge since nurses may change their responses in order to provide a more positive image of their smartphone use.

## Conclusions

The increasing utilization of smartphones in the nursing profession is recognized as a useful tool for improving communication and making better decisions when providing patient care. However, the study reveals nurses use their smartphones for personal usage and spend time on social media, which suggests a gap in their use of these tools for professional purposes. The potential for communication and decision-making at the point of care is enormous, but it's important to recognize the pitfalls of distraction and highlight the need for establishing environments that put patients' privacy and confidentiality first. The active participation of administration, nurses, and other healthcare providers is of the utmost importance in the development of a regulatory framework for smartphone use in the workplace. This collaborative effort ensures a digital code for conduct and efficient technological constraints customized to the specific needs and dynamics of the nurses regarding smartphone use in the workplace.
